# Risk Factors for Mortality in Melioidosis: A Single-Centre, 10-Year Retrospective Cohort Study

**DOI:** 10.1155/2021/8154810

**Published:** 2021-07-05

**Authors:** Raviraj Menon, Poornima Baby, Anil Kumar V., Sandeep Surendran, Manu Pradeep, Aiswarya Rajendran, Gaayathri Suju, Arathy Ashok

**Affiliations:** ^1^Department of General Medicine, Amrita Institute of Medical Sciences, Amrita Vishwa Vidyapeetham, Kochi, Kerala, India; ^2^Department of Microbiology, Amrita Institute of Medical Sciences, Amrita Vishwa Vidyapeetham, Kochi, Kerala, India; ^3^Department of Rheumatology, Amrita Institute of Medical Sciences, Amrita Vishwa Vidyapeetham, Kochi, Kerala, India; ^4^Department of Community Medicine, Amrita Institute of Medical Sciences, Amrita Vishwa Vidyapeetham, Kochi, Kerala, India; ^5^University of Massachusetts, Amherst, MA, USA; ^6^Amrita School of Medicine, Amrita Institute of Medical Sciences, Amrita Vishwa Vidyapeetham, Kochi, Kerala, India

## Abstract

Melioidosis is a tropical infectious disease with diverse clinical presentations. We aimed to investigate the characteristics and mortality risk factors of patients diagnosed with melioidosis in the past 10 years. This was a retrospective cohort study conducted at a quaternary care centre in South India. Clinical, demographic, and biochemical data in patients diagnosed with melioidosis with cultures were collected between January 2011 and December 2020 from medical records. Logistic regression analysis was performed to screen mortality risk factors of melioidosis in addition to descriptive statistics and chi-square analysis. Seventy-three melioidosis patients' records were analysed, and the most common comorbidity was type 2 diabetes mellitus (*n* = 53, 72.6%). The patients showed diverse presentations: pulmonary involvement, 30 (41.1%); splenomegaly, 29 (39.7%); abscesses and cutaneous involvement, 18 (24.7%); lymph node, 10 (13.7%); arthritis and osteomyelitis, 9 (12.3%); and genitourinary infection, 4 (5.4%). The mortality was noted to be 15 (20.5%). Logistic regression analysis indicated that chronic kidney disease (OR = 14.0), CRP >100 IU/L (OR = 6.964), and S. albumin <3 gm/dl (OR = 8.0) were risk factors associated with mortality and can guide in risk stratification. Hypoalbuminemia is a novel mortality risk factor, detected in this study, and requires further investigation to validate its utility as a prognostic marker and reveal possible therapeutic benefits in clinical correction.

## 1. Introduction

Melioidosis is an infection caused by the Gram-negative bacterium *Burkholderia pseudomallei*, which is a free-living organism found in surface water of rice paddies, freshly cultivated fields oil palm, drains, gardens, and playgrounds in endemic areas [1]. It is classified as a Class B Bioterrorism agent by the CDC and given the second highest priority [2]. Mode of transmission is commonly through direct human contact with infected soil or water, either by inhalation and cutaneous inoculation or by ingestion [3]. The leading risk factor for the disease is type 2 diabetes mellitus (DM), while other common risk factors include malignancy, chronic kidney disease, and immunosuppressive treatment [4, 5]. Clinical features differ considerably, from acute pulmonary or septicemic presentations that are often fatal, to chronic localized infection that can then worsen to acute sepsis, with *B. pseudomallei* cultured from blood, pus, urine, and other bodily tissues and secretions. Presentation can also vary, notably as a high fever, with abscesses in the lungs, spleen, and liver as well as bone and joint involvement [1]. According to a systematic review conducted in 2015, looking at clinical presentations of 8469 patients, pneumonia (35·7%), intra-abdominal abscess (18·3%), and sepsis (18%) were determined to be leading outcomes [6]. These confusing presentations of melioidosis demand diagnosis by microbiological confirmation [7]. High-sensitivity serological tests such as indirect hemagglutination assay (IHA), enzyme-linked immunosorbent assay (ELISA), and dot immunoassay have the potential to be used for screening for the infection in endemic communities but tissue culture remains the gold standard for diagnostic confirmation [8].

Melioidosis is a disease of alarming fatality, with overall mortality rates varying between 19 and 54% in different endemic regions [9]. The recommended antimicrobial therapy includes ceftazidime, trimethoprim/sulfamethoxazole, and doxycycline [1], but established melioidosis is hard to treat, leading to unprecedented patient demises [10]. A review of the published literature from cohorts in Australia and Thailand showed that age above 50 years, chronic kidney disease, and development of septicemia were predictors of mortality. A majority of melioidosis cases have been reported from North Eastern Thailand and North Australia, but it has been emerging steadily in the Indian subcontinent, China, the Middle East, Africa, and South America [11]. The west coast of India is particularly vulnerable as the climate, population density, and presence of risk factors among the populace favour endemicity [12]. Unfortunately, further data regarding epidemiology and risk factors for mortality in Indian patients are scanty since diagnosis and reporting are uncoordinated and sporadic. Hence, further investigation is warranted in Indian population.

The primary objective of this cohort was to study the clinical characteristics and biochemical markers and investigate for risk factors that may be associated with mortality in patients diagnosed with melioidosis in a quaternary care referral centre over the past 10 years.

## 2. Materials and Methods

### 2.1. Study Design and Inclusion and Exclusion Criteria

Designed as a retrospective cohort study, all patients who were diagnosed as having melioidosis at a quaternary care centre in the west coast of South India between January 1, 2011, and December 31, 2020, were considered. Patients were included in the study, if they were more than 18 years of age and had grown *B. pseudomallei* from culture samples done in the in-house microbiological laboratory. Patients were excluded, if their blood or tissue cultures were done by external laboratories or if there were insufficient clinical or demographic details in their medical records.

### 2.2. Identification of Isolates and Antimicrobial Susceptibility Testing

*B. pseudomallei* was grown from samples like blood, pus aspirate, tissue, sputum, pleural fluid, throat swab, synovial fluid, or urine as dry wrinkled oxidase positive colonies on 5% sheep blood agar and MacConkey agar media. On Gram staining, the organism appeared as a Gram-negative bacillus with bipolar staining. *B. pseudomallei* reduced nitrate, dihydrolysed arginine, and utilised glucose and lactose oxidatively. The isolates were further identified and the antimicrobial susceptibility testing was performed using the VITEK ®2 Compact system (bioMérieux).

### 2.3. Data Collection

All data were retrospectively compiled from the Electronic Medical Record (EMR) of the patients who satisfied the inclusion and exclusion criteria. Demographic data (age and gender) and comorbidities (DM, coronary artery disease, and hypertension, chronic kidney disease, malignancy) of all included participants were compiled. Clinical presentations were classified into groups by two authors to account for reporting bias, and in case of a dispute a third author's decision was used. Based on the primary organ affected at presentation, patient data was classified into (1) pulmonary: including pneumonia (radiological changes on X-ray or CT), pleural effusion, and lung abscess; (2) cutaneous: including soft tissue infections, and infections of nonskeletal tissue surrounding or supporting organs and other structures including subcutaneous tissue and muscle; (3) genitourinary infection of the genital organs and the urinary tract including the kidneys; (4) visceral abscesses; (5) osteomyelitis; and (6) septic arthritis, as well as splenomegaly and lymph nodal involvement. Additionally, key biochemical parameters were extracted and analysed including white blood cells (WBC) count, hemoglobin, erythrocyte sedimentation rate (ESR), C-reactive protein (CRP), albumin, and alkaline phosphatase. Mortality for each patient was also collected from death summaries from the EMR system. This study will acknowledge selection bias, as patient data is obtained from a quaternary care referral centre, but no correction is applied to address it.

### 2.4. Statistical Methods

Descriptive statistics included frequency analysis (percentages) for categorical variables and means ± standard deviations (SD) or median for continuous variables. Comparisons were determined by Student's *t*-test for continuous variables as appropriate and by the use of the *χ*2 test or Fisher exact test for categorical variables. Univariate logistic regression was performed to explore the association of significant parameters and estimate the mortality risk. The backward conditional method was used to select imaging variables entering the scoring system. The statistical significance level was set at 0.05 (two-tailed). All analyses were conducted with SPSS version 23.0 statistical software.

## 3. Results

This report describes 73 patients who were diagnosed with melioidosis at a quaternary care centre in South India between January 1st, 2011, and December 31st, 2020, after applying the inclusion and the exclusion criteria. Out of these, 65 (89%) needed inpatient care and 8 (11%) were managed on an outpatient basis. The mean (±SD) age was 49.55 ± 15.39 years. The median length of stay was 15 days (1–139 days). The basic clinical details along with the biochemical parameters are summarized in Table 1. Table 1 also shows a comparison of the present cohort with other South Indian cohorts of melioidosis [13, 14].

Seventy-one (97.2%) isolates of *B. pseudomallei* were found to be sensitive to ceftazidime, trimethoprim-sulfamethoxazole, chloramphenicol, and doxycycline. Sixty-one (83.5%) isolates were sensitive to imipenem, while sixty-two (85%) isolates were sensitive to tigecycline. The cohort was then stratified for mortality and analysed (Table 2). The three factors that showed significance underwent univariate analysis (Table 3). Chronic kidney disease (OR = 14), C-reactive protein >100 IU/L (OR 6.964), and S. albumin <3 gm/dl (OR = 8) were detected to be risk factors for mortality in patients.

## 4. Discussion

This retrospective cohort is based on data of patients diagnosed with melioidosis over the past 10 years in Amrita Institute of Medical Sciences, Kochi, a quaternary referral and training centre in South India. The clinical and epidemiological profile and mortality risk factors of patients diagnosed with melioidosis during this period were assessed and characterised. A total of 73 patients were diagnosed to have melioidosis, of which 65 patients were managed as inpatients in the hospital. Fifty-five patients (75.3%) were males with an average age of 49.5 ± 15.36 years at presentation, which was comparable with studies done by Saravu et al., 2010, and Currie et al., 2004 (Table 1). The reason for male preponderance may be explained by more frequent exposure to soil and water while in professions like farming or greater incidence of alcoholism among males [9, 15]. The most common comorbid condition was DM with 53 (72.6%) patients, followed by hypertension in 24 (32.8%) patients. The prevalence of DM detected in this study is comparable to those of other cohorts (74–80%) [7, 12, 16]. The average duration of symptoms on presentation ranged from 1 day to 4 months with a wide variety of clinical presentations noted within our cohort. Pulmonary involvement was the most common presentation with 30 cases (41.1%) which is comparable to lung involvement in Birnie et al., 2019 (35.7%), followed by splenomegaly, 29 (39.7%), abscesses and cutaneous involvement, 18 (24.7%), lymph node enlargement, 10 (13.7%), arthritis and osteomyelitis, 9 (12.3%) and genitourinary infection, 4 (5.4%) (Table 2). In contrast to sepsis reported by Birnie et al., 2019 (18%), this cohort reported a much higher 46.5% of patients presenting with sepsis. The median length of stay in hospital was 15 days (1–159 days).

The mortality in this cohort was noted to be 20.5%. The clinical presentation and mortality rates were comparable to those of a study done in Australia where the overall mortality was 19% with higher mortality in patients having CKD (31% of total deaths) [17]. In our study, logistic regression analysis indicated that chronic kidney disease, C-reactive protein >100 IU/L, and serum albumin <3 gm/dl were independent risk factors associated with mortality in melioidosis patients–(OR = 14.0, OR = 6.964, and OR = 8.0, respectively) (Table 3). This is in contrast to data from previous independent studies in Australia and Thailand where age more than 40 years, chronic kidney disease, occurrence of pneumonia, and septicemia were considered as predictors of mortality [18, 19].

Patients with chronic kidney disease have altered immune status with defective granulocyte, T-cells, and dendritic cell function, which predispose them to severe infection and increased mortality [20]. Low serum albumin levels result in increased mortality in patients with severe sepsis. This can be attributed to albumin being a carrier for several endogenous and exogenous molecules, with antioxidant and anti-inflammatory properties, and also it is the main protein involved in maintenance of plasma colloid osmotic pressure, all of which help to maintain hemodynamic stability. Albumin can also combine with free fatty acids in blood, which protects them from lipid peroxidation damage and it can also reduce or eliminate the toxicity of many exogenous toxic molecules by binding with them, which in turn reduces endothelial damage, as well as the risk of complications including mortality [21–23]. In contrast to the results of the present study, a 175-patient prospective study detected that CRP levels at admission were not found to be sensitive enough to categorise acute, chronic, or relapsed melioidosis in a suspected patient presenting with fever [24].

## 5. Conclusion

A limitation to note is that, as a quaternary care centre, with a greater number of referral cases from different hospitals, severity of the clinical outcome may show an institutional pattern which might not be applicable to population at large, thus incurring a selection bias. This may have led to an overestimation in the magnitude of impact of risk factors detected in the study, because the patient cases evaluated were of higher severity. Another limitation to note is the small sample size, but, relatively speaking, in context to the present Indian scenario, this study was analysed on a large patient data set with exhaustive biomarker records. The results discussed in this study may have external validity limited only to inpatient care in tertiary care centres, which remain the principal points of care in severe disease, equipped to diagnose and adequately manage melioidosis in India.

However, one should have a high index of suspicion for melioidosis and send appropriate cultures for the early diagnosis. Various risk factors for the disease should be identified at the earliest, and treatment should be initiated promptly after sending appropriate cultures. Patients having either chronic kidney disease or high CRP levels (more than 100 IU/l) or hypoalbuminemia could be identified as high-risk patients and treatment must be continued under close monitoring to pick up complications that may lead to mortality. Based on the results of this cohort, S. albumin may emerge as a novel independent modifiable risk factor with further evidence and may serve as a crucial prognostic parameter for risk stratification in melioidosis patients. Further clinical trials with S. albumin correction (target serum conc. of 3 gm/dl) as intervention can be safely tried in melioidosis patients presenting with sepsis. Patel et al., 2014, in a systematic review and meta-analysis, did not show a robust therapeutic benefit for human albumin in all-cause mortality of sepsis [25]. However, it is recommended that further large-scale trials be done to identify patient populations with microbiological evidence and build greater evidence with adequate analysis of biochemical markers associated with the high fatality rates of melioidosis associated sepsis.

## Figures and Tables

**Table 1:** Comparison between demographic, clinical, and laboratory findings of the present study against other single-centre South Indian cohorts.

Parameter	Present cohort	Saravu et al. [14]	Malladi et al. [13]
Number of patients	73	25	17
Region	Kerala	Karnataka > Kerala	Andhra Pradesh
Age in years	49.5 ± 15.36	47.9 ± 18.5	—

Gender			
Male	55 (75.3%)	15	13
Female	18 (24.7%)	10	4

Comorbidities			
DM	53 (72.6%)	19 (76%)	16 (94%)
Hypertension	24 (32.8%)	NA	—
Coronary artery disease	10	NA	—
Chronic kidney disease	7	3 (12%)	—
Malignancy	5	NA	—

Duration of symptoms	1 day to 4 months	2 days to 1 year	—

Bacteremia	34	13	12

Organ involvement			
Pulmonary	30 (41.1%)	9 (36%)	13 (76%)
Cutaneous	18 (24.7%)	—	10 (59%)
Genitourinary	4 (5.4%)	—	—
Arthritis	9 (12.3%)	3 (12%)	—
Osteomyelitis	9 (12.3%)	0	—
Abscess	18 (24.7%)	4 (16%)	7 (41%)
Lymph node	10 (13.7%)	2 (8%)	—
Splenomegaly	29 (39.7%)	—	—

Key biochemical parameters			
WBC count (× 10^3 cells/cu.mm)	13.52 ± 7.28	—	—
Hemoglobin (gm/dl)	10.78 ± 2.29	—	—
ESR (mm in the 1^st^ hr)	57.86 ± 22.618	—	—
CRP (IU/L)	126.46 ± 89.01	—	—
S. albumin (gm/dl)	4.47 ± 0.76	—	—
ALP (mg/dl)	159.51 ± 137.54	—	—

Mortality	15 (20.547%)	—	6 (35.294%)

**Table 2:** Demographic, clinical, and laboratory results stratified for mortality.

Parameter	Total (*n* = 73)	Not expired (*n* = 58)	Expired (*n* = 15)	*P* value
Sociodemographic variables				
Age	49.5 ± 15.36	50.95 ± 13.97	44.13 ± 19.48	0.219

Gender				0.639
Male	55	43	12	
Female	18	15	3	

Comorbidities				
DM	53	44	9	0.220
Hypertension	24	19	5	0.996
Coronary artery disease	10	7	3	0.419
Chronic kidney disease	7	2	5	0.003
Malignancy	5	3	2	0.271

Clinical and biochemical characteristics				
Duration of symptoms before diagnosis				0.712
More than 14 days	42	34	8	
Less than and equal to 14 days	31	24	7	

Organ involvement				
Pulmonary	30	21	9	0.095
Cutaneous	18	16	2	0.330
Genitourinary	4	2	2	0.185
Arthritis	9	8	1	0.675
Abscess	18	16	2	0.330
Osteomyelitis	9	9	0	0.189
Lymph node	10	10	0	0.110
Splenomegaly	29	25	4	0.549

Biochemical parameters				
Neutrophilic leucocytosis	27	24	3	0.147
ESR >70 mm/hr	17	11	6	0.086
CRP >100 IU/L	41	28	13	0.008
Albumin <3 gm/dl	29	26	3	0.004
ALP >110 mg/dl	41	32	9	0.737

**Table 3:** Univariate analysis of mortality risk factors in melioidosis.

Parameter	Univariate analysis	
	*P* value	Odds ratio (95% CI)
Chronic kidney disease	0.004	14.000 (2.379–82.398)
CRP >100 IU/L	0.016	6.964 (1.441–33.652)
S. albumin <3 gm/dl	0.010	8.000 (1.654–38.688)

## Data Availability

The patient medical records' data used to support the findings of this study have been deposited in the Mendeley Data repository (DOI: 10.17632/x7tvmxnb5t.1).
